# Limited observed efficacy of olfactory training in severe long-term post-COVID-19 syndrome: a prospective cohort study

**DOI:** 10.1007/s00405-026-10582-5

**Published:** 2026-09-07

**Authors:** Kathrin Kobus, Constantin Hintschich, Tomáš Palla, Theresa Griesbeck, Christopher Bohr, Veronika Vielsmeier

**Affiliations:** 1 Department of Otorhinolaryngology, University Hospital Regensburg, Franz-Josef-Strauß-Allee 11, D-93053 Regensburg, Germany; 2 Department of Ophthalmology, University Hospital LMU Munich, Munich, Germany; 3 Department of Infectiology and Infectious Diseases, University Hospital Regensburg, Regensburg, Germany

**Keywords:** Post-COVID syndrome, Olfactory dysfunction, Olfactory training, Quality of life, Anosmia

## Abstract

**Purpose:**

Persistent chemosensory dysfunction is a debilitating symptom of Post-COVID-19 Syndrome (PCS). While olfactory training (OT) is effective in early post-viral smell loss, its efficacy in chronic, multisystemic PCS remains unclear. This study aimed to characterize a severely affected cohort and to evaluate changes in olfactory function during a structured OT intervention.

**Methods:**

In this prospective study, 29 patients with persistent PCS lasting > 12 weeks (mean duration approximately 30 months) were recruited from a specialized post-COVID outpatient clinic. All patients underwent structured OT (standardized twice-daily exposure protocol) for 12 weeks. Participants underwent psychophysical testing (*Sniffin’ Sticks* TDI, Taste Strips) and completed questionnaires (self-MOQ, VAS, EQ-5D-5 L) before and after a 12-week OT period.

**Results:**

Of 25 patients included in the final analysis, 9 presented with psychophysical anosmia based on the TDI score. The cohort exhibited a high symptom burden, with health-related quality of life below population norms. Following OT, no statistically significant changes were observed in the objective TDI scores (19.0 ± 8.2 vs. 20.1 ± 10.5; *p* = 0.52). Similarly, subjective ratings (VAS) and questionnaires assessing olfactory quality of life showed no significant differences between baseline and follow-up. While individual transient improvements were reported, a persistant discrepancy between objective data and subjective perception was observed.

**Conclusion:**

Structured olfactory training was not associated with measurable improvement within the 12-week observation period in this cohort of chronic PCS patients. The lack of response may indicate that, in this severely affected subgroup, the effectiveness of olfactory training may be limited by factors beyond peripheral olfactory dysfunction, including reduced cognitive and functional capacity.

## Introduction

COVID-19 can lead to a broad spectrum of acute symptoms, the majority of which resolve spontaneously. Symptoms persisting for more than 4 weeks are classified as long-COVID, and those lasting minimum of 12 weeks are referred to as post-COVID syndrome (PCS) [1]. The clinical presentation is heterogeneous and may include fatigue, cognitive impairment, dyspnea, musculoskeletal pain, pain syndromes, psychological disturbances, and chemosensory dysfunction, often in complex combinations [2, 3].

While the exact pathophysiology of post-COVID syndrome remains incompletely understood, risk factors include female sex, older age, high body mass index and smoking history [4]. Previous hospitalization and intensive care admissions increase the likelihood of persistent symptoms [4–6], whereas vaccination against SARS-CoV-2 has been shown to decrease the incidence of post-COVID conditions [7]. Current management strategies remain largely symptomatic and supportive, with a strong focus on rehabilitation and functional restoration [8].

Chemosensory dysfunction is one of the most common manifestations of acute COVID-19 and may present as hyposmia, anosmia, hypogeusia, or qualitative alterations such as parosmia [9–11], while psychophysical studies suggest that true gustatory dysfunction may be less prevalent than subjectively reported [12]. Recovery of olfactory function occurs in approximately 90% of affected individuals within the first 3 months [13, 14], though 4–5% exhibit long-term or persistent deficits [15, 16]. Persistent dysfunction can significantly affect quality of life and heighten risk by impairing environmental hazard detection [17]. In patients with post-COVID syndrome, persistent olfactory dysfunction represents one component of a broader multisystemic condition that may additionally include fatigue, cognitive impairment, dyspnea, and chronic pain [1–3]. In the present study, olfactory training was specifically intended to target chemosensory dysfunction rather than the overall post-COVID symptom burden. Accordingly, the expected benefits of olfactory training were considered to be primarily olfactory rather than broadly systemic.

Olfactory training (OT) using standardized structured exposure to odorants has demonstrated therapeutic benefit across multiple etiologies of smell loss, including post-viral cases [18–20]. Recent systematic reviews in COVID-19-related olfactory dysfunction suggest that olfactory training may improve olfactory function, although results remain heterogeneous and depend on factors such as disease duration, training protocol, and patient characteristics [16, 20]. However, these findings are mainly derived from cohorts with early-phase post-viral olfactory dysfunction and relatively preserved general health status [21–23]. Evidence in patients with severe, long-standing and multisystemically impaired post-COVID syndrome remains limited, particularly for those with disease duration extending beyond the typical recovery window and undergoing olfactory rehabilitation [24]. Therefore, the objective of this study was to characterize a severely affected post-COVID outpatient cohort with long-term chemosensory impairment, representing a highly chronic and clinically burdened subgroup, and to evaluate changes in olfactory function during structured olfactory training using either standardized *Sniffin’ Sticks* or patient-selected natural odorants.

## Patients and methods

This prospective cohort study was conducted in accordance with the Declaration of Helsinki and approved by the Ethics Committee of the University of Regensburg (23-3531-101). Patients presenting to a specialized post-COVID outpatient clinic between March 1st, 2024, and December 31st, 2024, were screened for persistent sequelae of SARS-CoV-2 infection. Individuals reporting chemosensory symptoms were referred to the Department of Otorhinolaryngology for targeted evaluation.

In line with the study design, all eligible patients were offered structured olfactory training (OT) as part of standard clinical care. A non-intervention control group was not included due to ethical considerations in this highly symptomatic population.

Inclusion criteria comprised a subjective reduction of smell and/or taste for at least 12 weeks following a confirmed SARS-CoV-2 infection, established via PCR or rapid antigen testing. Patients were required to participate in allocated olfactory training for the full duration of the study. Exclusion criteria included age < 18 years, absence of chemosensory self-report, known alternative causes of olfactory loss, or inability to complete follow-up.

Baseline metrics included demographics, time from SARS-CoV-2 infection to presentation, duration of chemosensory impairment, number of SARS-CoV-2 infections, vaccination status (number of doses), health-related quality of life assessed by the EuroQol 5-Dimension 5-Level questionnaire (EQ-5D-5 L; higher scores indicate better health status) and the 36-Item Short Form Health Survey for General Health (SF-36; higher scores indicate better health status), fatigue assessed by the Fatigue Scale for Motor and Cognitive Functions (FSMC; higher scores indicate greater fatigue), pain intensity, occupational status (including sick leave), current medication, comorbidities, allergies, and nicotine use. All data were pseudonymized prior to analysis.

Psychophysical and subjective chemosensory function was assessed using the *Sniffin’ Sticks* threshold–discrimination–identification (TDI) test, the 16-item Taste Strips test and the self-reported mini olfactory questionnaire (self-MOQ) [25]. Taste function was additionally assessed to provide a comprehensive characterization of chemosensory dysfunction and its longitudinal course in this patient population, given the close relationship between olfaction and flavor perception, while not representing a direct therapeutic target of olfactory training although no direct effect of olfactory training on gustatory function was anticipated. Patients additionally rated their olfactory function using a visual analogue scale (VAS; 0 = minimum, 10 = maximum). Nasal endoscopy and examination of the oral cavity was performed in all cases.

Patients were allocated to 2 training interventions: (i) standardized odor training using commercially available *Sniffin’ Sticks*, or (ii) individualized training using patient-selected natural odorants. In the latter approach, participants were provided with a list of example substances but were free to select alternative odorants according to personal preference. They were instructed to use four distinct odorants and to replace them after 4 and 8 weeks. The most frequently chosen natural scents for the OT were cinnamon, vinegar, thyme, and coffee. In contrast to standardized odor sets, these natural odorants may vary in composition and concentration, allowing for a more individualized training approach. The use of both standardized and patient-selected odorants reflects different approaches used in clinical practice; however, the study was not designed to formally compare these interventions.

Olfactory training was performed according to a structured, video-assisted protocol as originally proposed by Hummel et al. [18]. Participants were instructed to train twice daily (morning and evening), with focused exposure to each odorant for approximately 10 s and interstimulus intervals. Odor sets were changed after 4 and 8 weeks. Adherence to olfactory training was assessed by patient self-report at follow-up visits; no standardized or objective adherence measures were implemented. Follow-up evaluation was conducted after 12 weeks, including repeated TDI, gustatory testing, and Self-MOQ. The VAS was additionally re-assessed by telephone after 4 and 8 weeks. No additional treatments were administered during the olfactory training period.

Patients with long-standing PCS and chemosensory impairment, including clinical severity, symptom duration, and quality-of-life impact were characterized. The primary objective was the psychophysical improvement or deterioration of smell or taste, measured using the TDI score and the 16-item Taste Strips test. The secondary endpoint was subjective improvement assessed via the VAS (embedded in the self-MOQ) as well as changes in self-MOQ scores. Tertiary endpoints included the correlation of health-related quality of life (HRQoL), fatigue and the self-MOQ with the olfactory improvement (ΔTDI). Improvement was defined as the change in outcome measures from baseline to the 12-week follow-up within subjects without implying causality of the intervention due to the study design. Between-group comparisons were considered exploratory and were not predefined as primary or secondary endpoints, and no causal inference regarding intervention efficacy was intended due to the absence of a control group.

Statistical analyses were performed using Python (version 3.13) with Pandas, NumPy, SciPy and statsmodels; figures were generated using Matplotlib. Continuous data are presented as median [interquartile range] or mean ± standard deviation (SD). Significance was set at *p* < 0.05. Pre- and post-training measures were compared using the Wilcoxon signed-rank test; longitudinal VAS ratings were analysed using linear mixed-effects models with a random intercept for participant. Associations were assessed using Spearman rank correlations using complete cases per analysis, with nonparametric percentile bootstrap 95% confidence intervals. Agreement between self-MOQ- and TDI-based diagnostic categories was quantified using quadratically weighted Cohen’s κ.

## Results

### Post-COVID-19 syndrome and chemosensory impairment

**Fig. 1:**
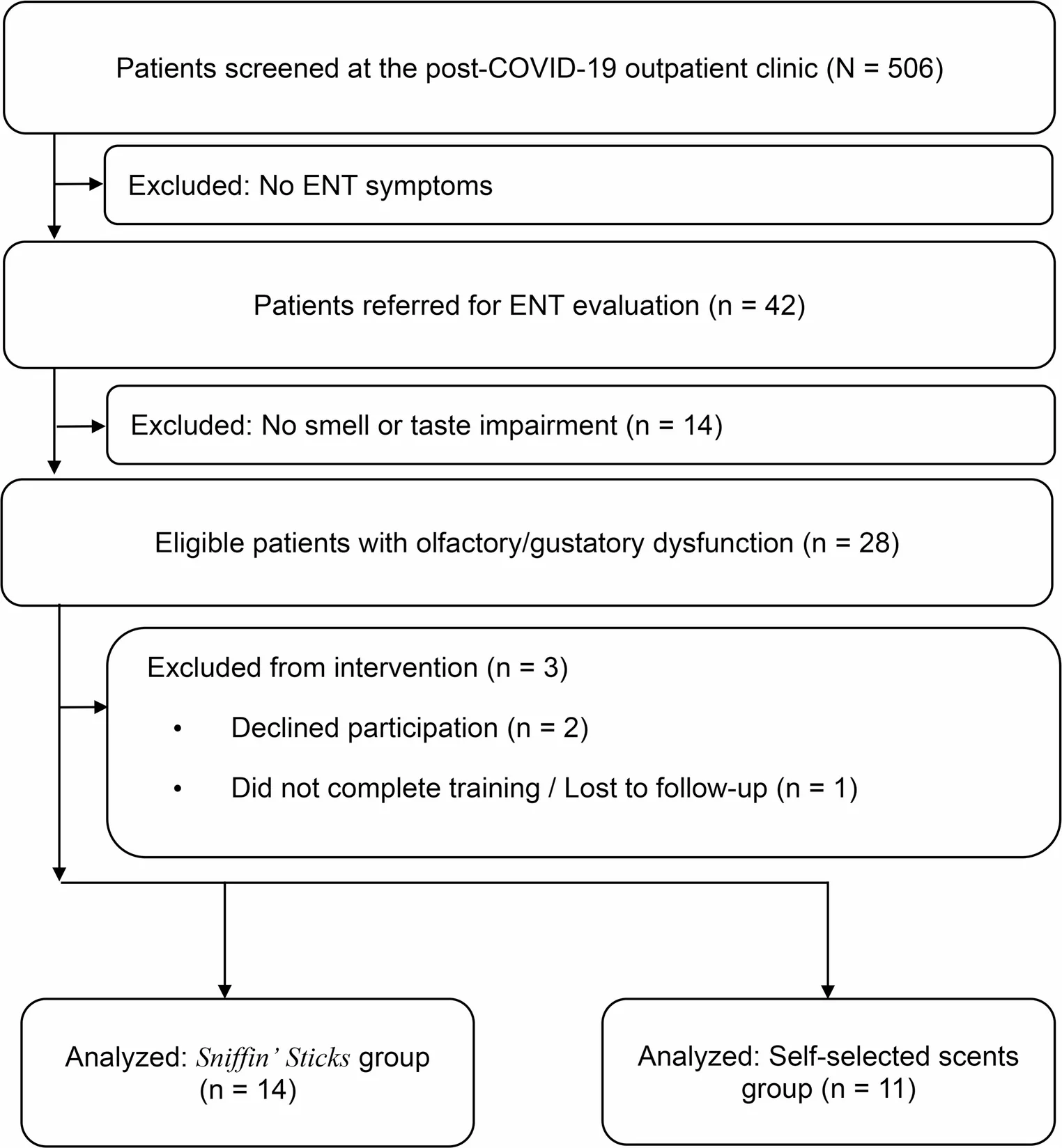
Patient flow chart. Illustration of the screening, inclusion, and analysis process. N, n: number of patients

A total of 506 patients presented at the post-COVID-19 outpatient clinic, of whom 42 were evaluated by the ENT department for persistent otorhinolaryngological symptoms (Fig. 1). These included a range of complaints such as tinnitus, hearing loss, and chemosensory dysfunction. Among these, 29 patients (mean age 52.5 ± 13.7 years, 69% male) reported impaired smell and/or taste. 1 patient reported onset of olfactory loss following COVID-19 vaccination and was excluded from further analysis. Of the remaining 28 patients, 26 patients initiated the training protocol, while 2 declined participation. 1 patient was lost to follow-up. Consequently, a total of 25 patients were included in the final efficacy analysis, comprising 14 patients in the *Sniffin’ Sticks* group and 11 patients in the self-selected odorant group.

In the analyzed cohort, olfactory dysfunction coincided with the onset of the acute COVID-19 infection. Patients had experienced a mean of 2.0 ± 1.1 SARS-CoV-2 infections and had received 2.5 ± 0.9 vaccinations; 1 was unvaccinated. 7 patients were active smokers. The mean interval between symptom onset and first consultation at the post-COVID-19 outpatient clinic was 29.6 ± 12.2 months. Qualitative olfactory dysfunction (parosmia) was reported by 17 patients.

General health status and quality of life were notably impaired. The median EQ-5D-5 L VAS score was 50 [10; 80] (German normative mean 71.59 ± 21.36, [26]), with a corresponding median Index (Utility) score of 0.72 [-0.12; 0.93] (German normative mean 0.88 ± 0.18, [26]). Similarly, the median SF-36 General Health score was 35 [5; 95] (German normative mean 69.3; 95% CI 68.7–69.9) [27]). Evaluation of fatigue via the FSMC revealed severe fatigue in 18 patients and moderate fatigue in 3 patients. Moreover, the cohort exhibited a high somatic and socioeconomic burden, with 20 patients reporting persistent pain and 15 currently on sick leave. Baseline demographic, clinical, and patient-reported characteristics are summarized in Table 1.

**Table 1:** Demographic characteristics and longitudinal outcomes of psychophysical and subjective assessments

	Total Cohort	Patient-Selected OT	OT with *Sniffin’ Sticks*
	*n* = 25	*n* = 11	*n* = 14
Age (years)	51.3 ± 13.7	50.9 ± 12.9	51.6 ± 14.6
Male sex (%)	72	55	86
Nasal polyps, n	2	1	1
EQ Index (median)	0.72 [-0.12; 0.93]	0.62 [0.25; 0,76]	0.72 [0.30; 0,73]
EQ VAS (median)	50 [10; 80]	46 [40; 66]	50 [42; 60]
SF-36 (median)	35 [5; 95]	37,5 [21; 59]	32,5 [26; 40]
TDI (Baseline)	19.0 ± 8.2	19.6 ± 9.0	18.5 ± 7.9
TDI (Week 12)	20.1 ± 10.5	19.5 ± 10.2	20.5 ± 11.1
p-value	0.52	0.83	0.33
anosmia (T0/ T3)	9 / 11	4 / 5	5 / 6
hyposmia (T0/ T3)	14 / 7	5 / 3	9 / 4
normosmia (T0/ T3)	2 / 7	2 / 3	0 / 4
Taste (Baseline)	6.2 ± 4.1	6.6 ± 4.0	5.9 ± 4.4
Taste (Week 12)	7.2 ± 4.7	8.1 ± 4.5	6.4 ± 5.0
p-value	0.11	0.19	0.34
VAS (Baseline)	2.7 ± 2.3	2.9 ± 2.7	2.5 ± 2.0
VAS (Week 4)	3.2 ± 2.2	2.9 ± 2.1	3.4 ± 2.4
VAS (Week 8)	3.2 ± 2.1	3.0 ± 2.1	3.4 ± 2.2
VAS (Week 12)	2.4 ± 2.1	1.9 ± 1.9	2.7 ± 2.2
p-value	0.37	0.11	0.57
self-MOQ (Baseline)	4.0 ± 1.4	4.2 ± 1.3	3.9 ± 1.5
self-MOQ (Week 12)	4.2 ± 1.3	4.5 ± 1.0	4.1 ± 1.5
p-value	0.14	0.25	0.39

Data are presented as mean ± standard deviation (*SD*), unless otherwise indicated as median [interquartile range] (e.g., for EQ-5D, SF-36). Intra-group comparisons (baseline vs. 12 weeks) were calculated using the Wilcoxon signed-rank test and for VAS ratings the linear mixed-effects models. *OT* Olfactory Training, *TDI* Composite score of Threshold, Discrimination, and Identification (*Sniffin’ Sticks*), *VAS* Visual Analog Scale (0–10), *self-MOQ* Self-reported Mini Olfactory Questionnaire, *T0* Baseline, *T1* 4 weeks, *T2* 8 weeks, *T3* 12 weeks (final assessment). *n* Number of patients

### Visual analogue scale (VAS)

**Fig. 2:**
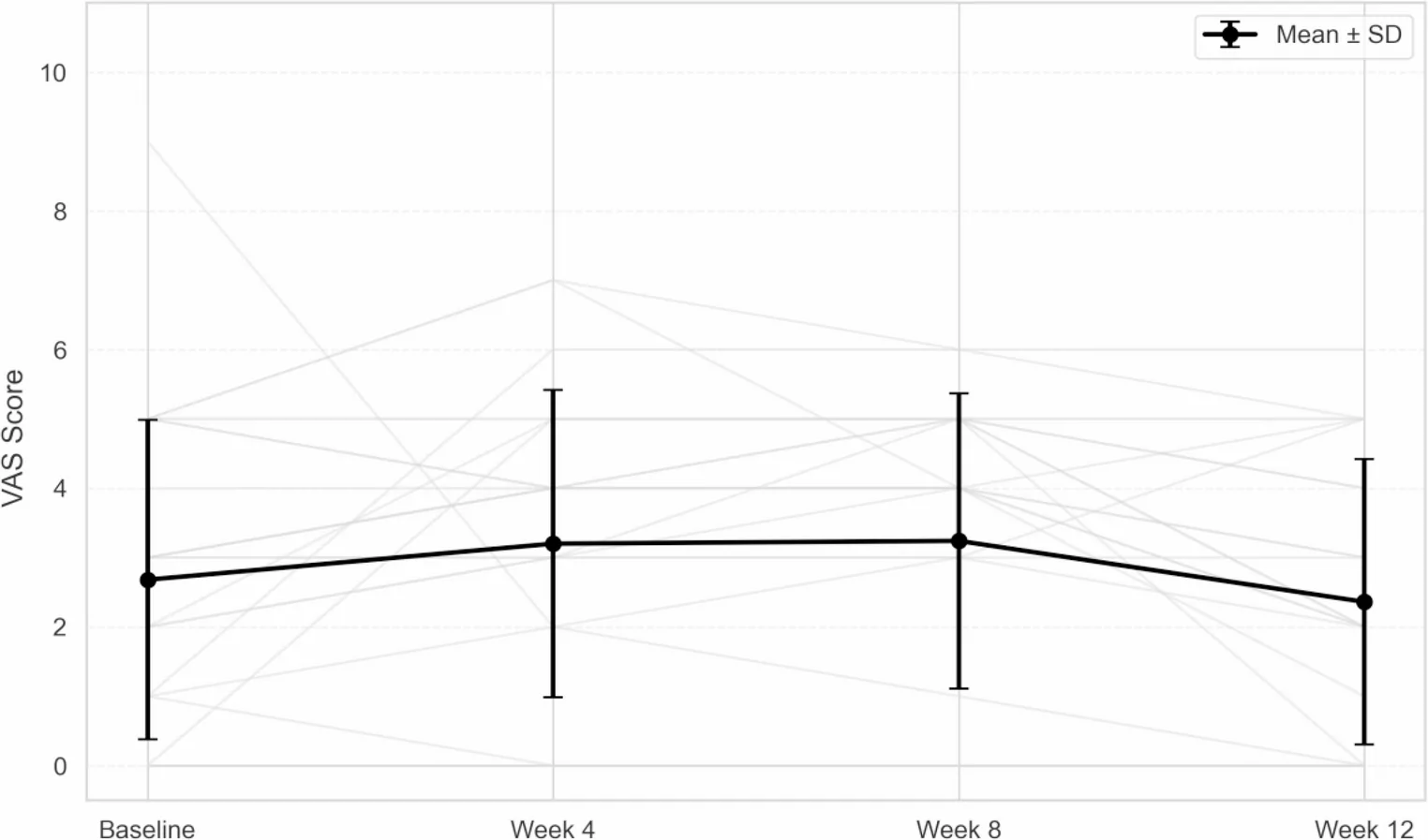
Longitudinal course of subjective olfactory function. Visual Analogue Scale (VAS) scores were assessed at baseline, and after 4, 8, and 12 weeks of training. Grey lines represent individual patient trajectories, illustrating high inter-individual variability. The black line indicates the mean ± standard deviation (SD)

Mean VAS scores (0 = minimum, 10 = maximum) increased from 2.7 ± 2.3 at baseline to 3.2 ± 2.2 at week 4 and 3.2 ± 2.1 at week 8, before decreasing to 2.4 ± 2.1 at week 12 (Fig. 2). In the overall linear mixed-effects model (random intercept for participant), VAS did not change significantly over time (all contrasts vs. baseline p > 0.05). In exploratory subgroup-specific mixed-effects models, the *Sniffin’ Sticks* group showed higher VAS at weeks 4 and 8 compared with baseline (*p* = 0.013 for both), whereas no significant changes were observed in the odorants group. In the combined model including a time-by-group interaction, there was no evidence that VAS trajectories differed between training modalities (week 4 × group *p* = 0.19; week 8 × group *p* = 0.23; week 12 × group *p* = 0.08).

### Self-MOQ

The Self-MOQ assesses subjective recognition of everyday odors, with scores > 4.5 indicating anosmia, 3.5–4.5 hyposmia, and < 3.5 normosmia. Mean scores showed no significant change, shifting from 4.0 ± 1.4 at baseline to 4.2 ± 1.3 after training (*p* = 0.14). Among the 25 patients, 14 met criteria for anosmia and 4 for hyposmia at baseline. After the training period, 17 patients met the criteria for anosmia and 2 for hyposmia; the remaining patients were classified as normosmic.

### Psychophysical olfactory testing (TDI)

**Fig. 3:**
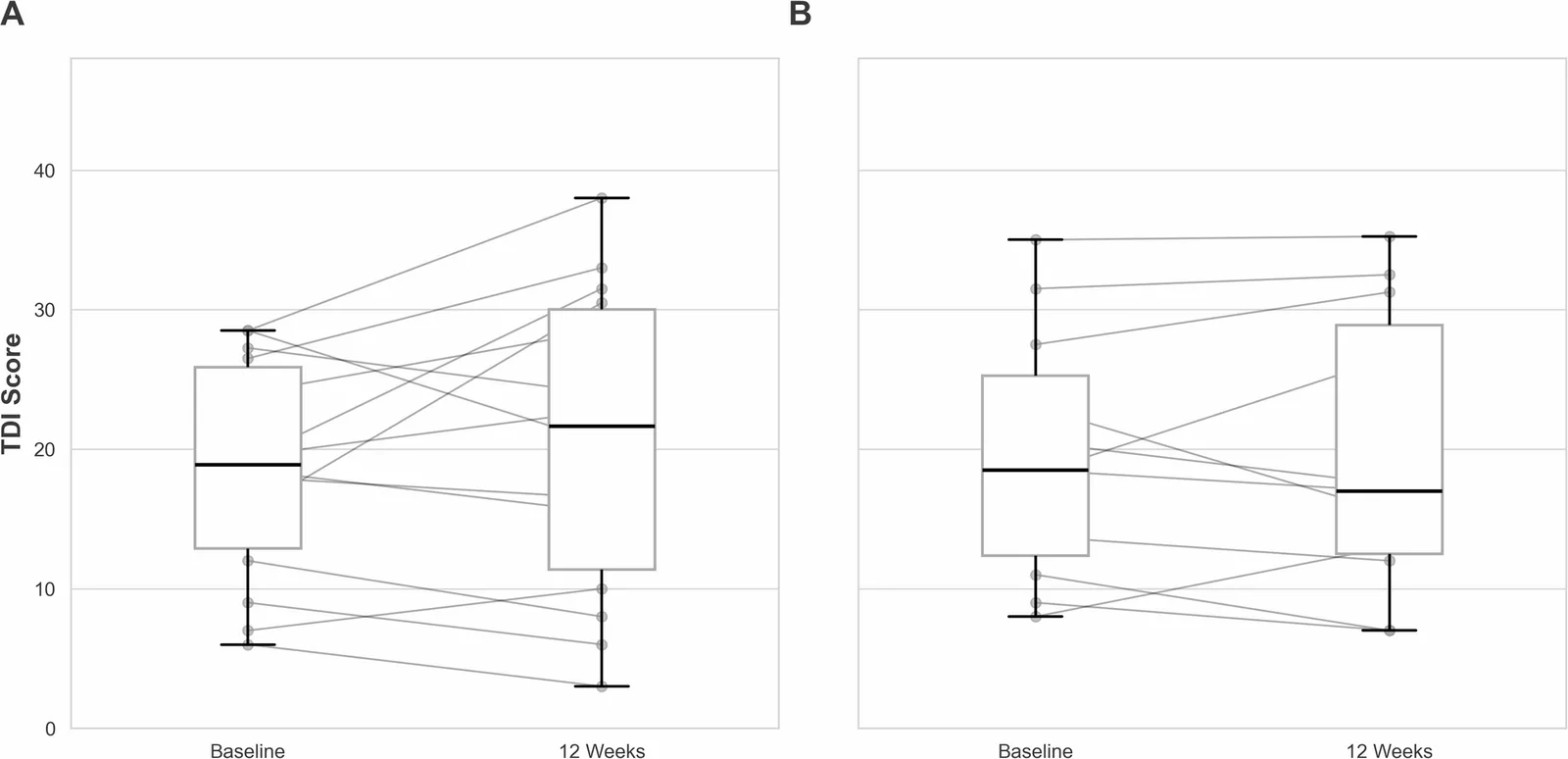
Changes in olfactory function (TDI score) after 12 weeks. (A) Standardized training group (*Sniffin’ Sticks*). (B) Patient-selected odorant group. Boxplots show median and IQR; grey lines indicate individual trajectories

The composite TDI score (sum of threshold, discrimination, and identification scores) was calculated to assess overall olfactory function. Mean TDI scores increased from 19.0 ± 8.2 at baseline to 20.1 ± 10.5 after 12 weeks of training; however, this change was not statistically significant (p = 0.52). No significant pre-to-post changes were observed within either the *Sniffin’ Sticks* group (*p* = 0.33) or the self-selected scents group (*p* = 0.83) (Fig. 3).

Regarding clinically relevant changes (defined as ≥ 5.5 points), 5 patients demonstrated improvement, while 2 patients showed deterioration (Table 2) [28].

**Table 2:** Patients with a change in TDI-score of more than 5.5 points

Patient number	Smell training	Patient delay	TDI before	TDI after	TDI difference	Taste test before	Taste test after	VAS before	VAS 4 weeks	VAS 8 weeks	VAS 12 weeks	EQ Index
1	*Sniffin’ Sticks*	27.4	15.5	30.5	**15**	8	7	1	6	6	6	0.79
2	*Sniffin’ Sticks*	25.9	19.25	31.5	**12.25**	8	9	5	7	6	5	0.75
3	*Sniffin’ Sticks*	23.5	28.5	38	**9.5**	11	14	5	4	5	5	0.72
4	*Own scents*	6.0	18.25	26.5	**8.25**	7	9	5	5	5	0	0.65
5	*Sniffin’ Sticks*	22.3	26.25	33	**6.75**	3	7	5	4	5	4	0.73
6	*Sniffin’ Sticks*	27.7	28.5	20.5	**–8**	4	2	2	3	3	3	0.93
7	Own scents	29.2	23	15.5	**–7.5**	6	12	0	5	5	2	0.62

*TDI* score, Threshold-Discrimination-Identification-score. *VAS* visual analog scale. Patient delay in month

Based on established cut-offs (TDI ≤ 16.5: anosmia; 16.5 < TDI < 30.5: hyposmia; ≥ 30.5: normosmia), 9 patients presented with anosmia, 14 with hyposmia, and 2 with normosmia at baseline. After the 12-week training period, the number of normosmic patients increased to 7, while those with hyposmia decreased to 7. The number of patients with anosmia remained relatively stable (*n* = 11). Comparable distribution patterns were observed in both training subgroups.

### Correlation of HRQoL/ fatigue with ΔTDI

In correlation analyses restricted to patients who completed olfactory testing at baseline and week 12 and had non-missing HRQoL/ fatigue measures (*n* = 24), baseline EQ-5D-5 L index, EQ-VAS, SF-36 General Health, and FSMC total score were not significantly associated with olfactory improvement (ΔTDI). Effect estimates were small and imprecise (Spearman ρ = 0.25 for EQ-5D-5 L index; ρ = −0.08 for EQ-VAS; ρ = 0.29 for SF-36 General Health; ρ = 0.04 for FSMC; all *p* ≥ 0.17), and all bootstrap 95% confidence intervals included 0. Exploratory subgroup analyses by training modality showed similarly wide confidence intervals; within the natural odorants subgroup, SF-36 General Health exhibited a moderate positive correlation with ΔTDI (ρ = 0.60), but this did not reach statistical significance (*p* = 0.065) and remained imprecise (bootstrap 95% CI including zero).

### Subjective - objective concordance (self-MOQ vs. TDI)

At baseline, self-MOQ scores (higher = worse subjective olfaction) showed a strong inverse association with psychophysical olfactory function (TDI; higher = better function) (Spearman ρ = −0.69, *p* < 0.001; bootstrap 95% CI − 0.84 to − 0.43; *n* = 25). At week 12, the correlation was weaker and did not reach statistical significance (ρ = −0.38, *p* = 0.062; bootstrap 95% CI − 0.79 to 0.13). Changes over time were not concordant: Δself-MOQ was not associated with ΔTDI (ρ = 0.17, *p* = 0.42; bootstrap 95% CI − 0.27 to 0.53).

Agreement between self-MOQ- and TDI-based categorical classifications (anosmia/ hyposmia/ normosmia) was moderate at baseline (quadratically weighted Cohen’s κ = 0.57; *n* = 25) and decreased to fair–moderate at week 12 (κ = 0.39; *n* = 25), indicating only partial concordance between subjective categorization and psychophysical testing. These findings suggest that subjective and objective measures capture related but distinct dimensions of olfactory dysfunction.

### Gustatory function (16-item taste strips)

Mean cumulative taste scores (range 0–16) increased from 6.2 ± 4.1 at baseline to 7.2 ± 4.7 after the training period. This change was not statistically significant (p = 0.11). Subgroup analysis similarly revealed no significant improvements for either the self-selected scents group (p = 0.19) or the *Sniffin’ Sticks* group (*p* = 0.34).

## Discussion

In the present study, only minimal changes in psychophysical olfactory and gustatory function were observed over the 3-month follow-up period. Given the absence of a non-intervention control group, these findings should be interpreted descriptively, and spontaneous recovery or regression to the mean cannot be excluded. This finding contrasts with prior investigations that demonstrated measurable improvements following structured olfactory training (OT) in patients with post-viral olfactory dysfunction [20–23]. A key distinguishing factor in our cohort is the latency between infection and intervention. While olfactory regeneration appears to be most robust in the early post-infectious phase, any potential efficacy may decline as the duration of dysfunction increases. With a mean latency of nearly 30 months between the initial COVID-19 infection and study inclusion, our cohort represents a markedly long-standing symptom duration as a negative prognostic factor for olfactory rehabilitation [29–32]. This may suggests that the potential for neuroplastic recovery could be significantly diminished in cases of chronic, long-term dysfunction.

Crucially, however, the lack of improvement may not be solely attributable to chronicity but also to the specific morbidity of the cohort. Previous research has predominantly focused on otherwise healthy individuals presenting with isolated smell loss and mild systemic symptoms [20–23]. In stark contrast, our patients suffered from a severe, multi-systemic PCS [1, 2], characterized by pronounced fatigue, chronic pain, and long-term occupational disability. In this context, olfactory impairment is embedded within a complex neurocognitive and somatic symptom cluster [1–3]. It is therefore conceivable that this systemic burden could be associated with reduced effectiveness of olfactory training, although this cannot be established in the present study. Since successful olfactory training relies on top-down cognitive processing and sustained attention, its effects are thought to be mediated by experience-dependent neuroplasticity within central olfactory networks and by attentional and multisensory mechanisms rather than peripheral regeneration alone [33, 34]. Thus, cognitive fatigue and impaired attention in severe PCS may potentially reduce effective training exposure and blunt rehabilitation-related plasticity. However, this interpretation remains hypothetical and cannot be directly inferred from the present data.

A further important consideration is patient adherence to the training protocol. Adherence was assessed based on patient self-report, with all participants indicating that they had performed the training as instructed. However, no standardized or objective adherence measures (e.g., training diaries or digital monitoring) were implemented. Given the pronounced fatigue, cognitive impairment, and overall symptom burden in this cohort, reduced or inconsistent adherence cannot be excluded and may have influenced the observed outcomes. This constitutes a relevant potential confounder and may have contributed to the limited changes observed.

This hypothesis of a depleted physiological and psychological reserve is substantiated by our analysis of health-related quality of life (HRQoL). To account for the data distribution, we report median values, which reveal a profound impairment deviating substantially from population norms. While the German normative mean for the EQ-5D-5 L Index is 0.88 ± 0.18 [26], our cohort presented a markedly lower median of 0.72 [-0.12; 0.93]. 22 of 25 patients (88%) scored below this population average. This pattern is paralleled in the EQ-VAS and SF-36 General Health scores, confirming a widespread deficit [26, 27]. However, baseline HRQoL and fatigue measures were not significantly associated with subsequent olfactory improvement over 12 weeks (ΔTDI). Correlation estimates were small and accompanied by wide bootstrap confidence intervals, indicating considerable uncertainty and suggesting that, within this limited sample, baseline HRQoL did not reliably predict training-related olfactory change. Effect sizes were small and imprecise, with confidence intervals including zero, indicating substantial statistical uncertainty. While an exploratory signal of a positive association between SF-36 General Health and ΔTDI was observed in the natural odorants subgroup, this finding did not reach statistical significance and should be interpreted cautiously given the small subgroup size and imprecision. Overall, these data suggest that although systemic burden is substantial in this population, its relationship to short-term olfactory change may be complex and potentially mediated by unmeasured factors (e.g., qualitative chemosensory symptoms, cognitive constraints, or adherence).

Furthermore, the persistent distress underscores a notable discrepancy we observed between subjective self-ratings and psychophysical test results. Our data provide quantitative support for a dissociation between subjective olfactory experience and psychophysical performance in this severely affected PCS cohort. While self-MOQ correlated strongly with TDI at baseline, the association weakened at week 12 and changes in self-MOQ did not track changes in TDI over time. In addition, agreement between self-MOQ- and TDI-derived diagnostic categories was only moderate at baseline and decreased further at follow-up, underscoring that subjective classification and psychophysical testing capture overlapping but non-identical constructs. This subjective-objective dissociation has been described in post-COVID cohorts [35], and is further supported by the symptom profile of our cohort of our study group (19 of 28 patients reported parosmia). It is crucial to note that standard psychophysical tools like the TDI primarily measure olfactory *function* (threshold and identification) but are blind to such *qualitative* distortions. Consequently, patients may correctly identify an odor (yielding higher TDI scores) while perceiving it as distorted or aversive in daily life. This high prevalence of parosmia explains why the ‘normosmia’ label masked a clinically relevant impairment in our cohort. Furthermore, the persistent subjective burden implies that for patients with severe PCS, this qualitative deficit remains a major source of anxiety and reduced quality of life, aligning with the bidirectional relationship described by Croy et al. [17].

The interpretation of our findings is subject to several limitations. First, the relatively small sample size and the absence of a non-training control group substantially restrict causal inferences regarding the specific efficacy of olfactory training, as spontaneous recovery and regression to the mean cannot be excluded. Second, although 2 different olfactory training approaches were applied, the study was not designed or powered to formally compare these groups, and any between-group analyses must therefore be considered exploratory. Third, adherence was not objectively measured and may represent an additional source of bias. Fourth, due to the cohort’s heterogeneity regarding comorbidities and symptom trajectories, a detailed subgroup stratification was not feasible. Fifth, a potential practice effect cannot be entirely ruled out for the group using *Sniffin’ Sticks*, as the training set included 3 odors (lemon, clove, and rose) that also serve as items in the TDI identification test. Sixth, selection bias is likely, as participants were recruited from a tertiary care setting and represented a highly symptomatic subgroup. Finally, psychophysical assessments may have been influenced by fluctuating motivation or cognitive fatigue (performance bias) [17], and the 3-month observation period may have been insufficient to capture delayed recovery trajectories in this very long-term chronic population [36]. Future controlled studies are needed to disentangle intervention-specific effects from natural recovery in this patient population. Accordingly, the present findings should be interpreted as descriptive and hypothesis-generating.

## Conclusion

In conclusion, our findings indicate that while olfactory training is an established intervention for early post-viral dysfunction in otherwise healthy individuals, it may have limited rehabilitative value in patients with severe, chronic PCS. The lack of improvement suggests that in this highly chronic and systemically impaired population,

the effectiveness of olfactory training may be limited in this cohort, potentially due to factors such as reduced cognitive reserve and sustained attentional impairment, although this cannot be directly inferred from the present data. Future therapeutic strategies should therefore consider integrating systemic and neurocognitive rehabilitation approaches alongside sensory training.
